# Supplementary material for: The prediction of swim start performance based on squat jump force-time characteristics
Source: PeerJ. 2020 Jun 1;8:e9208. doi: 10.7717/peerj.9208 (PMC7271885; doi:10.7717/peerj.9208)
Supplement: Table S1 [file peerj-08-9208-s001.docx]

Supplementary Table 1: Definition of squat jump variables obtained from the ForceDecks force platform.

| **Variable** | **Definition** |
| --- | --- |
| Concentric impulse [N.s.] | Net impulse of vertical force during the Concentric Phase |
| Concentric mean force [N] | Mean vertical force during the concentric phase |
| Concentric mean power [W] | Mean power during concentric phase |
| Concentric peak force [N] | Peak vertical force during the concentric phase |
| Concentric rate of power development (RPD) [W/s] | Rate of power development between start of concentric phase to peak power |
| Force at peak power [N] | Vertical force at moment of peak power |
| Peak power [W] | Maximum power in the concentric phase |
| Reactive strength index modified (RSImod) [m/s] | Jump height (Flight Time) divided by contraction time |
| Take-off peak force [N] | Maximum vertical force over from start of movement to take-off |
| Concentric peak velocity [m/s] | Peak velocity during concentric phase |
| Concentric rate of force development (RFD) BW [N/s/kg] | Rate of force development for vertical force during the concentric phase divided by body mass |
| Concentric RFD [N/s] | Rate of force development for vertical force during the concentric phase |
| Jump height (impulse-momentum) [cm] | Jump height calculated by taking velocity at the instant of take-off and predicting the maximum vertical displacement of the centre of mass based on body mass (measured in centimetres) |
| Velocity at peak power [m/s] | Velocity at peak power (from start of movement to take-off) |
